# Supplementary figures and images for: Deep imaging reveals dynamics and signaling in one-to-one pollen tube guidance
Source: EMBO Rep. 2024 May 21;25(6):6. doi: 10.1038/s44319-024-00151-4 (PMC11169409; doi:10.1038/s44319-024-00151-4)

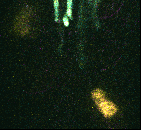

Supplement: Supplementary file 5 — Source data Fig. 1 [file 44319_2024_151_MOESM5_ESM.zip › Figure1/1F/1F_xy_z-projection_stacks.tif]

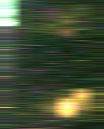

Supplement: Supplementary file 5 — Source data Fig. 1 [file 44319_2024_151_MOESM5_ESM.zip › Figure1/1G/1G_yz_x-projection_stacks.tif]

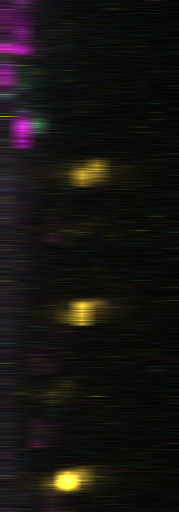

Supplement: Supplementary file 5 — Source data Fig. 1 [file 44319_2024_151_MOESM5_ESM.zip › Figure1/1E/1E_yz_x-projection_stacks.tif]

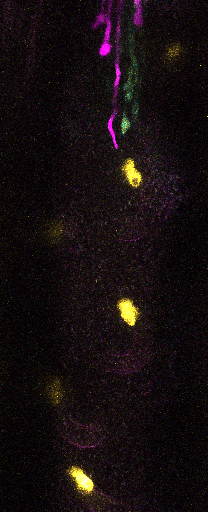

Supplement: Supplementary file 5 — Source data Fig. 1 [file 44319_2024_151_MOESM5_ESM.zip › Figure1/1D/1D_xy_z-projection_stacks.tif]

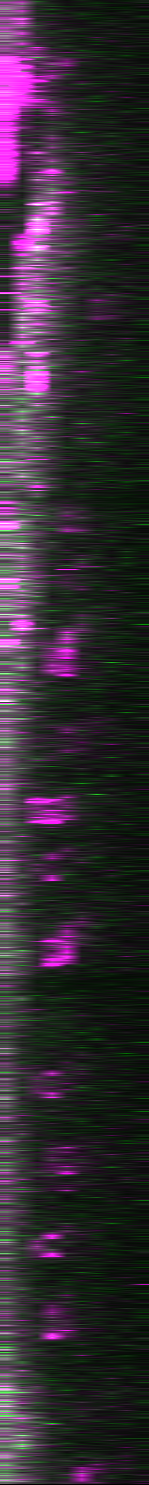

Supplement: Supplementary file 5 — Source data Fig. 1 [file 44319_2024_151_MOESM5_ESM.zip › Figure1/1C/1C_yz_x-projection_stacks.tif]

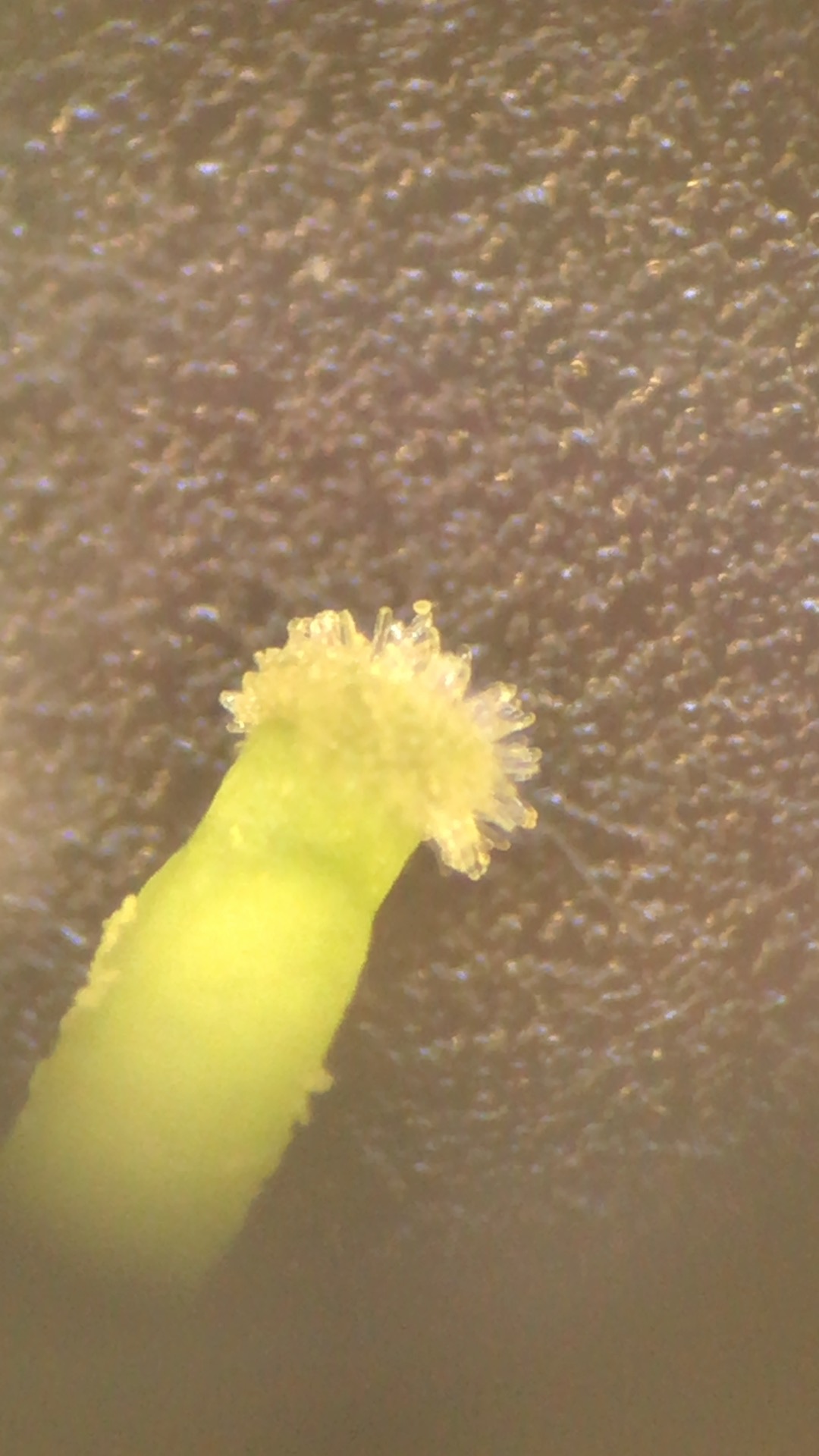

Supplement: Supplementary file 6 — Source data Fig. 2 [file 44319_2024_151_MOESM6_ESM.zip › Figure2/2A/2A.JPG]

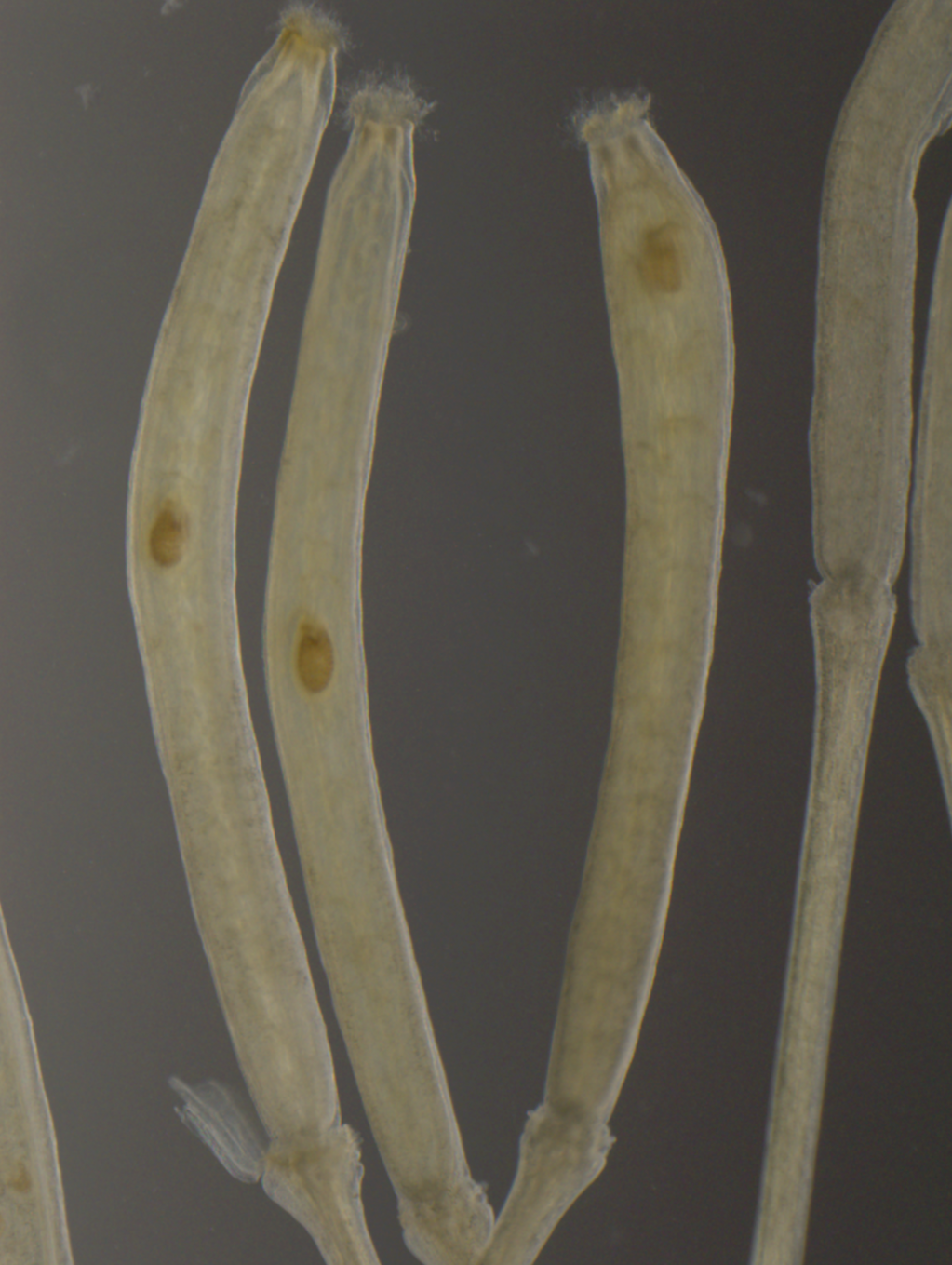

Supplement: Supplementary file 6 — Source data Fig. 2 [file 44319_2024_151_MOESM6_ESM.zip › Figure2/2C/2C.tif]

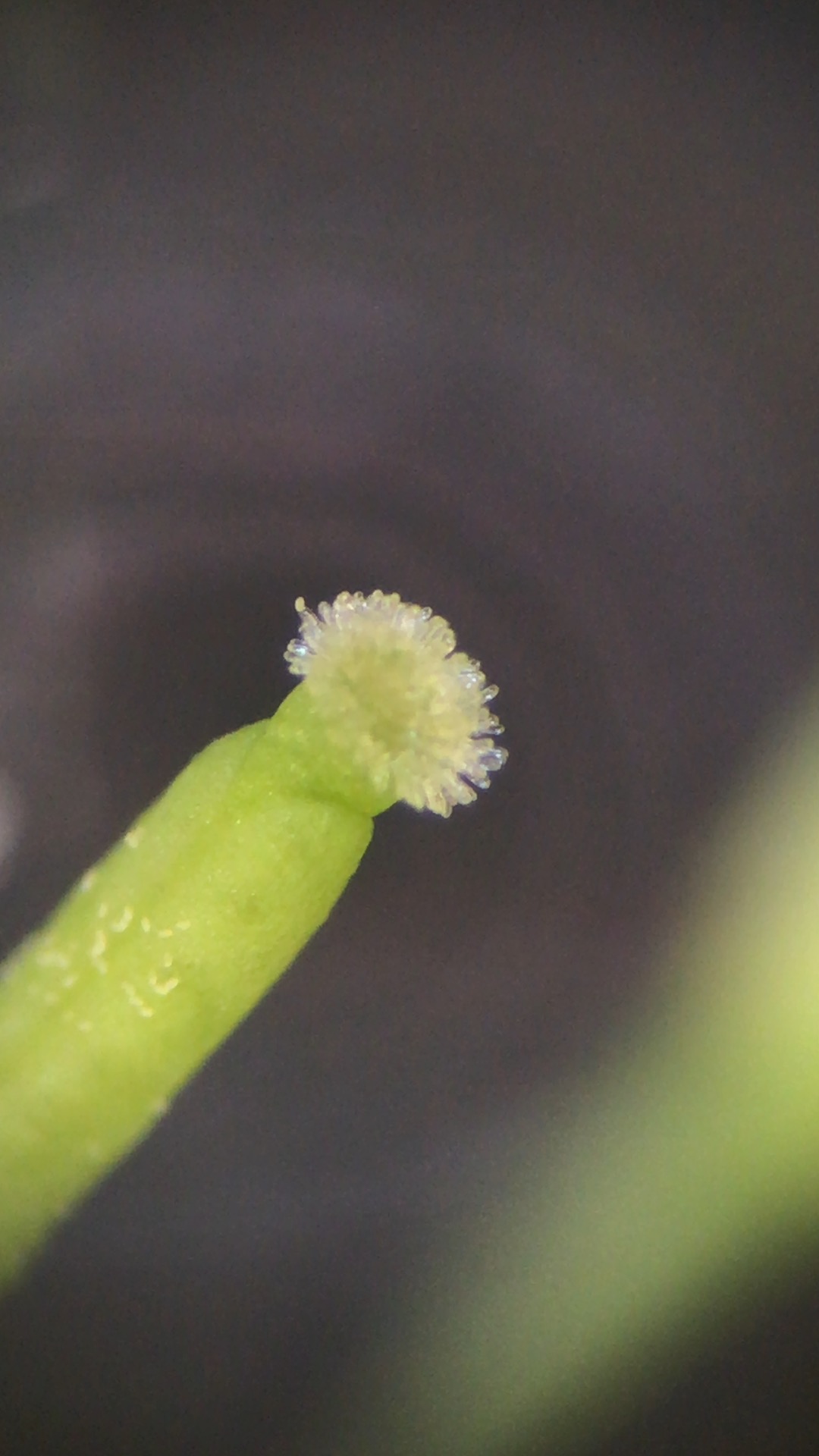

Supplement: Supplementary file 6 — Source data Fig. 2 [file 44319_2024_151_MOESM6_ESM.zip › Figure2/2B/2B.JPG]

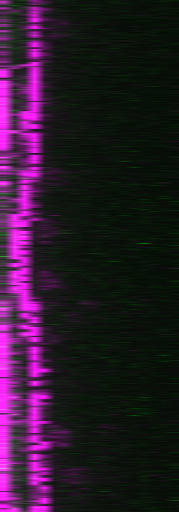

Supplement: Supplementary file 7 — Source data Fig. 3 [file 44319_2024_151_MOESM7_ESM.zip › Figure3/3B/3B_x-projection_crop_stacks.tif]

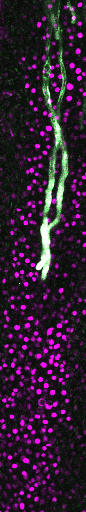

Supplement: Supplementary file 7 — Source data Fig. 3 [file 44319_2024_151_MOESM7_ESM.zip › Figure3/3C/3C_z-projection_crop_stacks.tif]

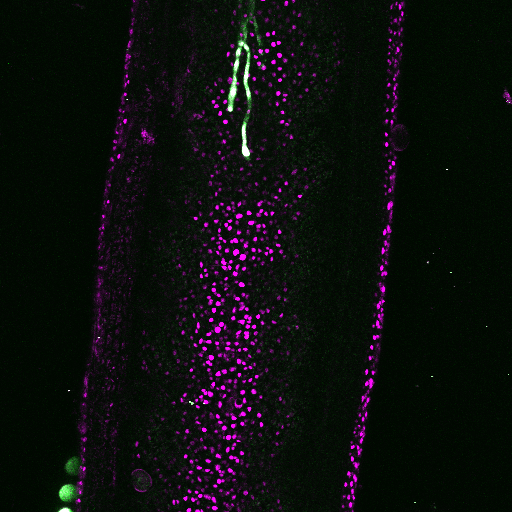

Supplement: Supplementary file 7 — Source data Fig. 3 [file 44319_2024_151_MOESM7_ESM.zip › Figure3/3C/3C_z-projection_whole_stacks.tif]

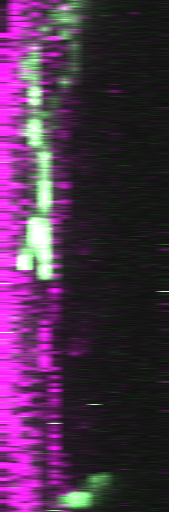

Supplement: Supplementary file 7 — Source data Fig. 3 [file 44319_2024_151_MOESM7_ESM.zip › Figure3/3D/3D_x-projection_crop_stacks.tif]

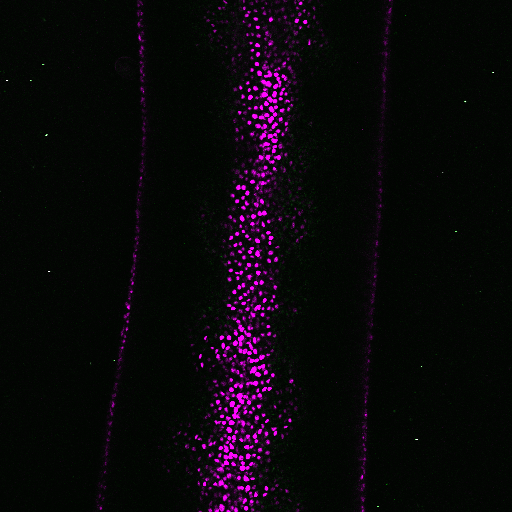

Supplement: Supplementary file 7 — Source data Fig. 3 [file 44319_2024_151_MOESM7_ESM.zip › Figure3/3A/3A_z-projection_whole_stacks.tif]

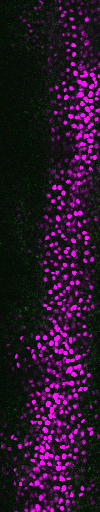

Supplement: Supplementary file 7 — Source data Fig. 3 [file 44319_2024_151_MOESM7_ESM.zip › Figure3/3A/3A_z-projection_crop_stacks.tif]

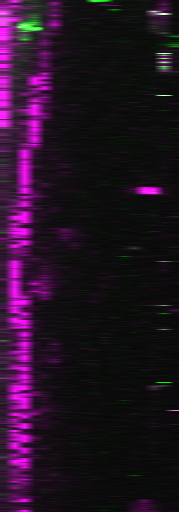

Supplement: Supplementary file 8 — Source data Fig. 4 [file 44319_2024_151_MOESM8_ESM.zip › Figure4/4B/4B_x-projection_stacks.tif]

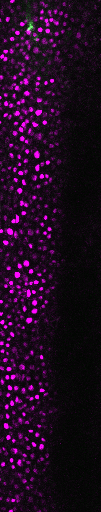

Supplement: Supplementary file 8 — Source data Fig. 4 [file 44319_2024_151_MOESM8_ESM.zip › Figure4/4B/4B_z-projection_crop_stacks.tif]

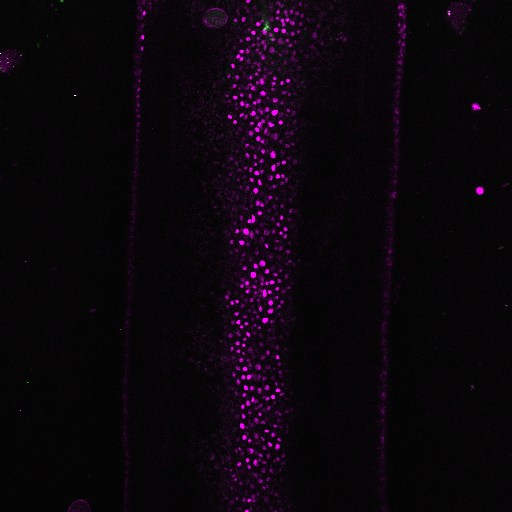

Supplement: Supplementary file 8 — Source data Fig. 4 [file 44319_2024_151_MOESM8_ESM.zip › Figure4/4B/4B_z-projection_whole_stacks.tif]

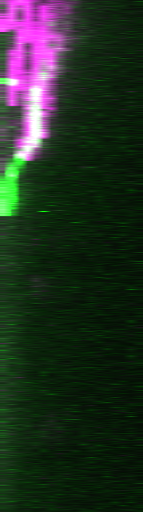

Supplement: Supplementary file 8 — Source data Fig. 4 [file 44319_2024_151_MOESM8_ESM.zip › Figure4/4D/4D_x-projection_crop_stacks.tif]

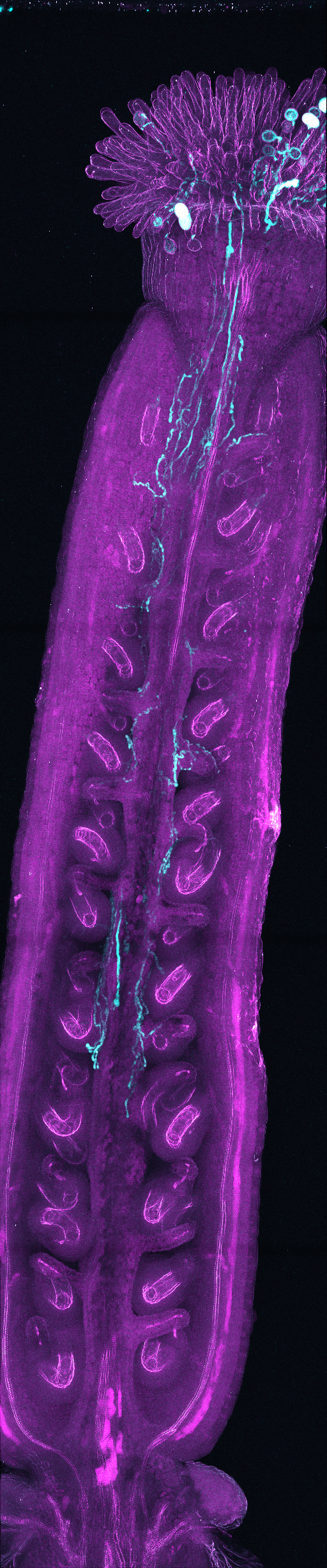

Supplement: Supplementary file 9 — Source data Fig. 5 [file 44319_2024_151_MOESM9_ESM.zip › Figure5/5A/5A_wt.tif]

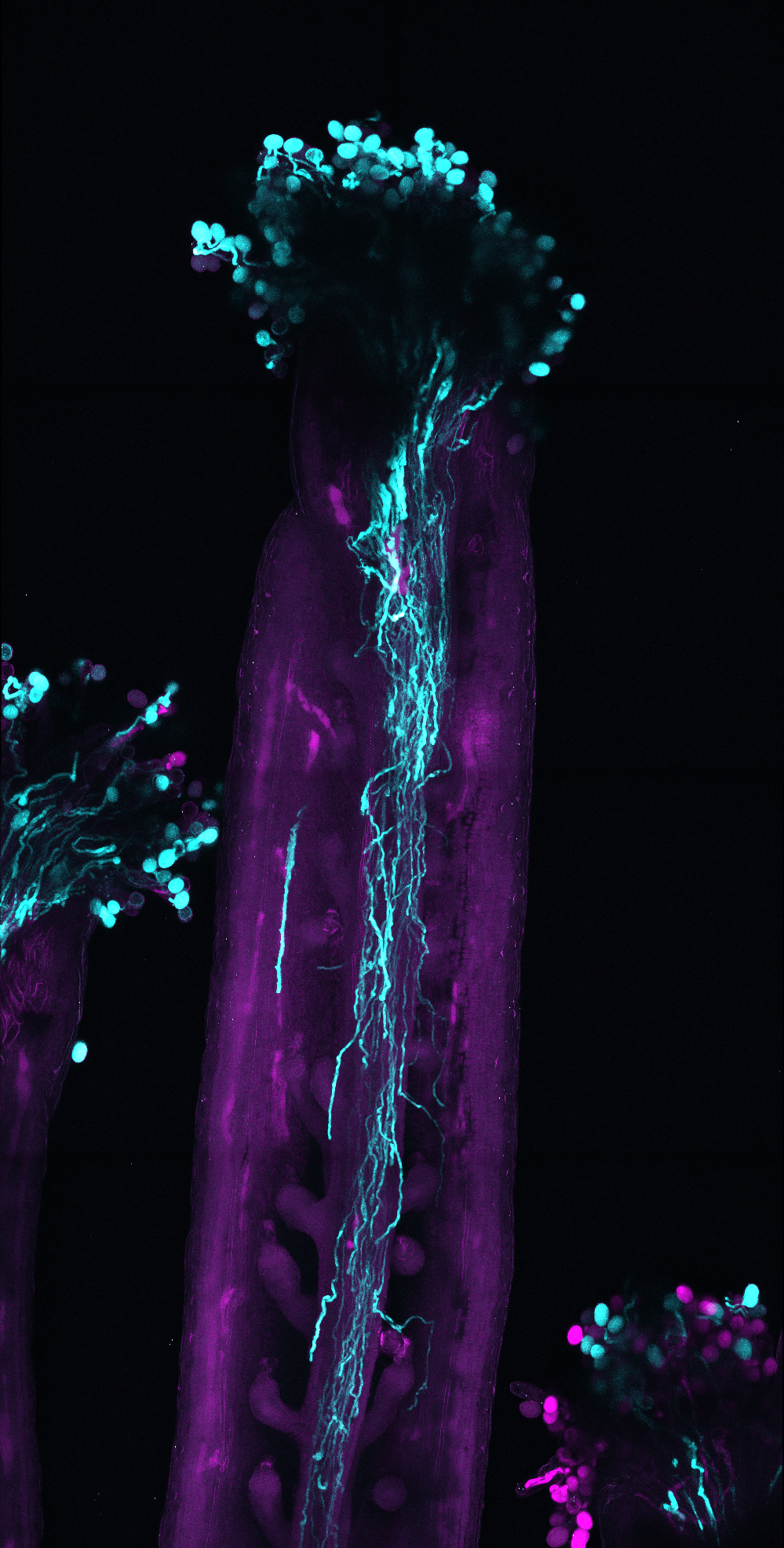

Supplement: Supplementary file 9 — Source data Fig. 5 [file 44319_2024_151_MOESM9_ESM.zip › Figure5/5A/5A_ant.tif]

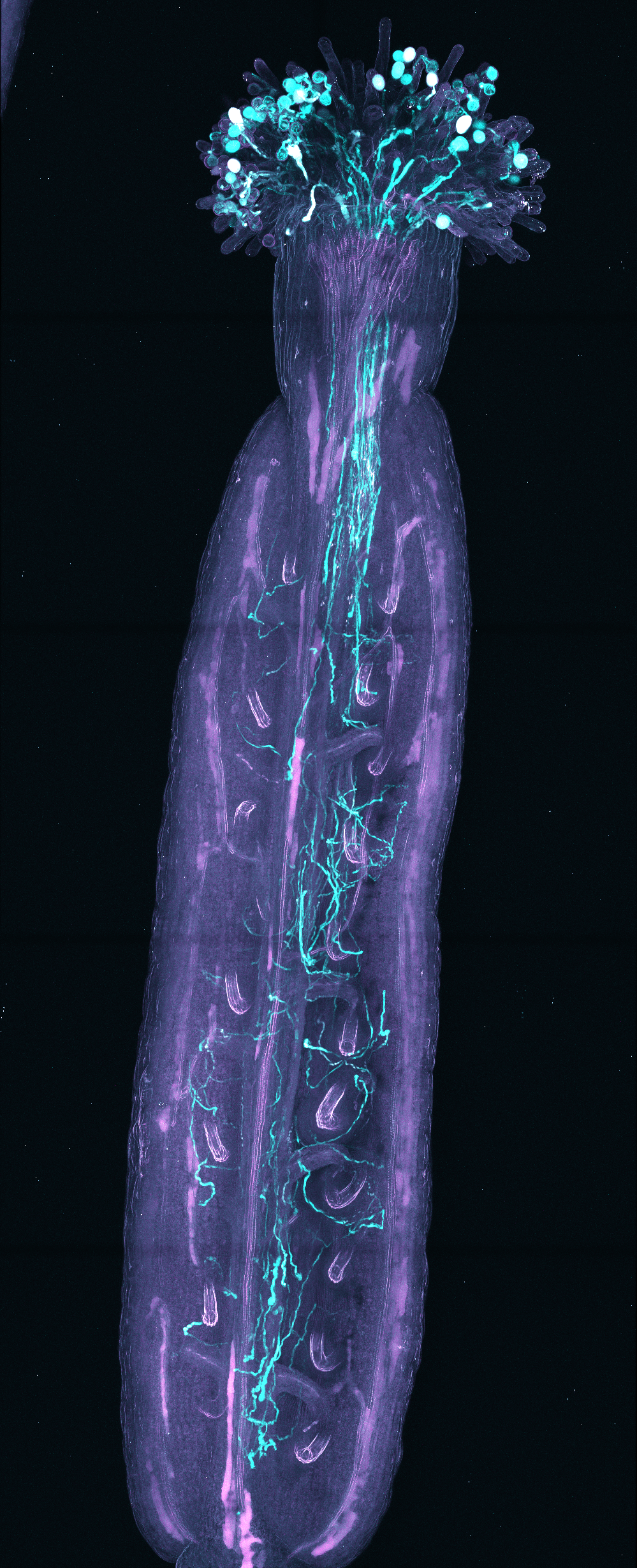

Supplement: Supplementary file 9 — Source data Fig. 5 [file 44319_2024_151_MOESM9_ESM.zip › Figure5/5A/5A_dif1.tif]

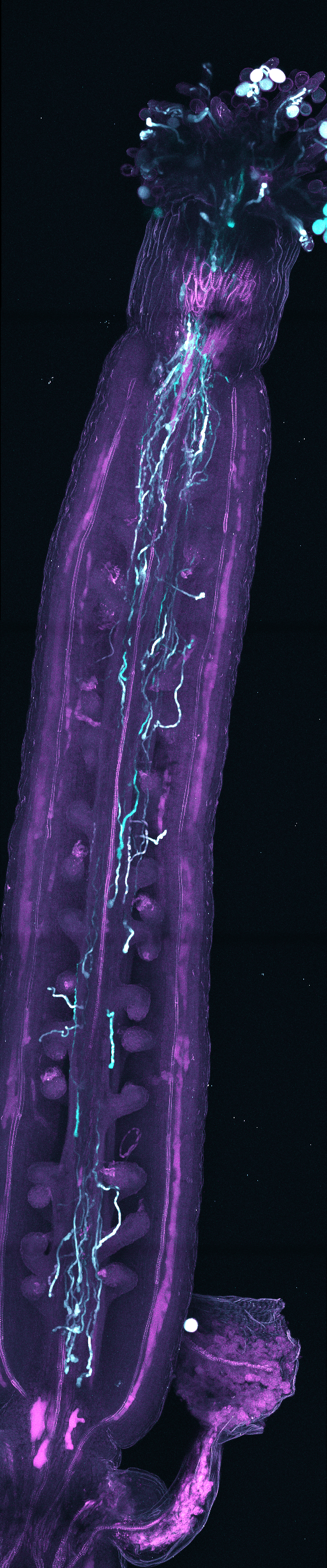

Supplement: Supplementary file 9 — Source data Fig. 5 [file 44319_2024_151_MOESM9_ESM.zip › Figure5/5A/5A_ino.tif]

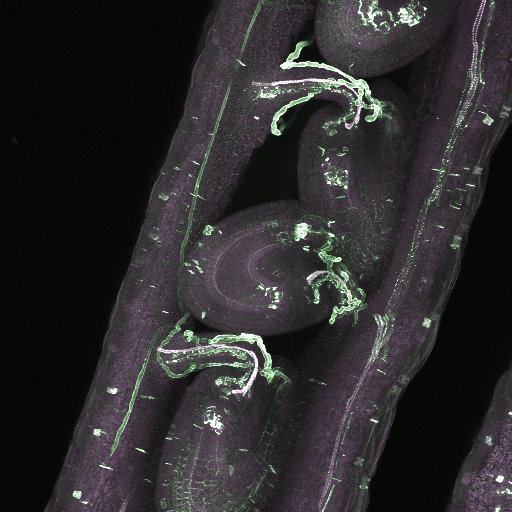

Supplement: Supplementary file 10 — Source data Fig. 6 [file 44319_2024_151_MOESM10_ESM.zip › Figure6/6A/6A_lre.tif]

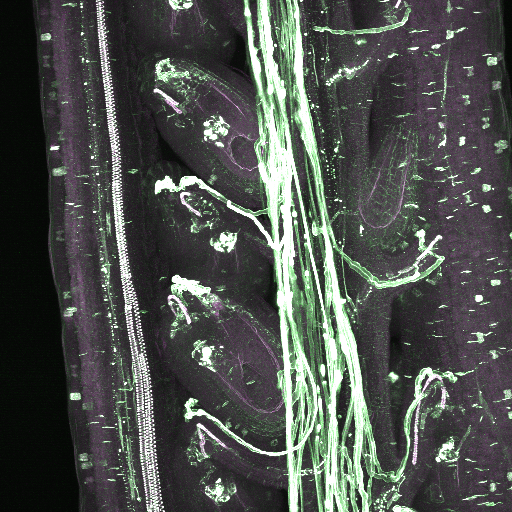

Supplement: Supplementary file 10 — Source data Fig. 6 [file 44319_2024_151_MOESM10_ESM.zip › Figure6/6A/6A_gcs1.tif]

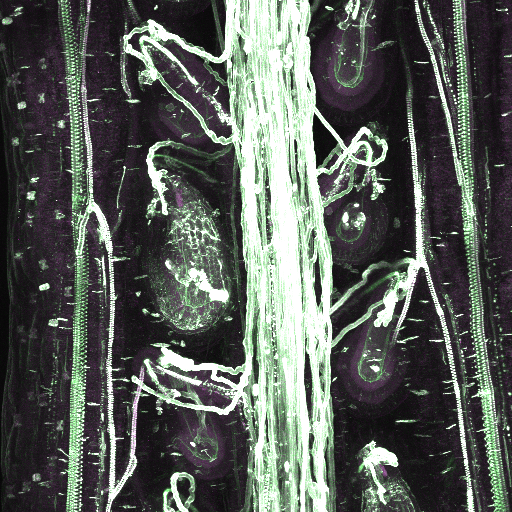

Supplement: Supplementary file 10 — Source data Fig. 6 [file 44319_2024_151_MOESM10_ESM.zip › Figure6/6A/6A_fer.tif]

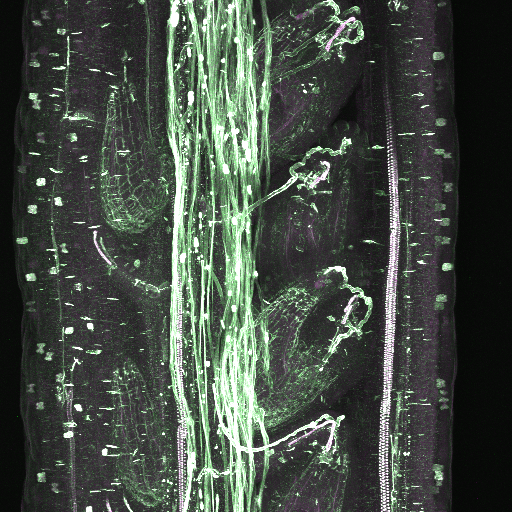

Supplement: Supplementary file 10 — Source data Fig. 6 [file 44319_2024_151_MOESM10_ESM.zip › Figure6/6A/6A_WT.tif]

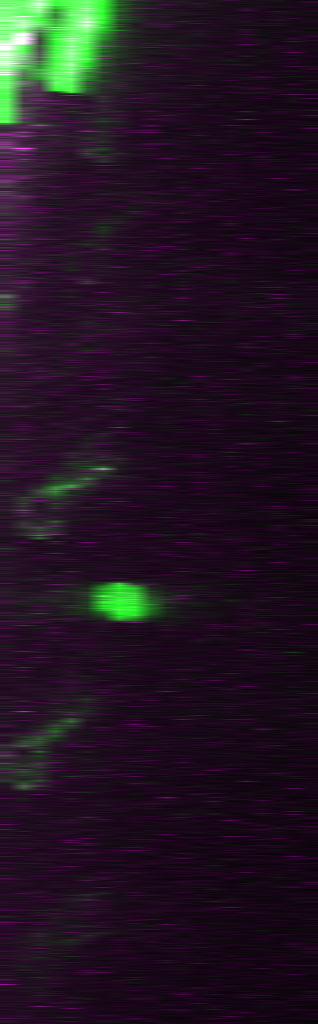

Supplement: Supplementary file 11 — Source data Fig. 7 [file 44319_2024_151_MOESM11_ESM.zip › Figure7/7C/7C_lre_x-projection_stacks.tif]

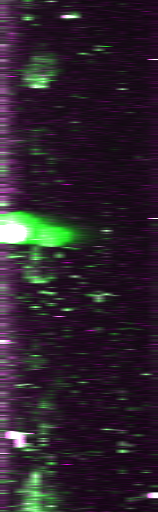

Supplement: Supplementary file 11 — Source data Fig. 7 [file 44319_2024_151_MOESM11_ESM.zip › Figure7/7B/7B_fer_x-projection_stacks.tif]

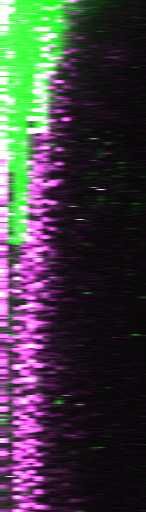

Supplement: Supplementary file 11 — Source data Fig. 7 [file 44319_2024_151_MOESM11_ESM.zip › Figure7/7A/7A_WT_x-projection_stacks.tif]
