# Supplementary material for: Deep imaging reveals dynamics and signaling in one-to-one pollen tube guidance
Source: EMBO Rep. 2024 May 21;25(6):6. doi: 10.1038/s44319-024-00151-4 (PMC11169409; doi:10.1038/s44319-024-00151-4)
Supplement: Supplementary file 13 — Expanded View Figures [file 44319_2024_151_MOESM13_ESM.pdf]

Expanded View Figures

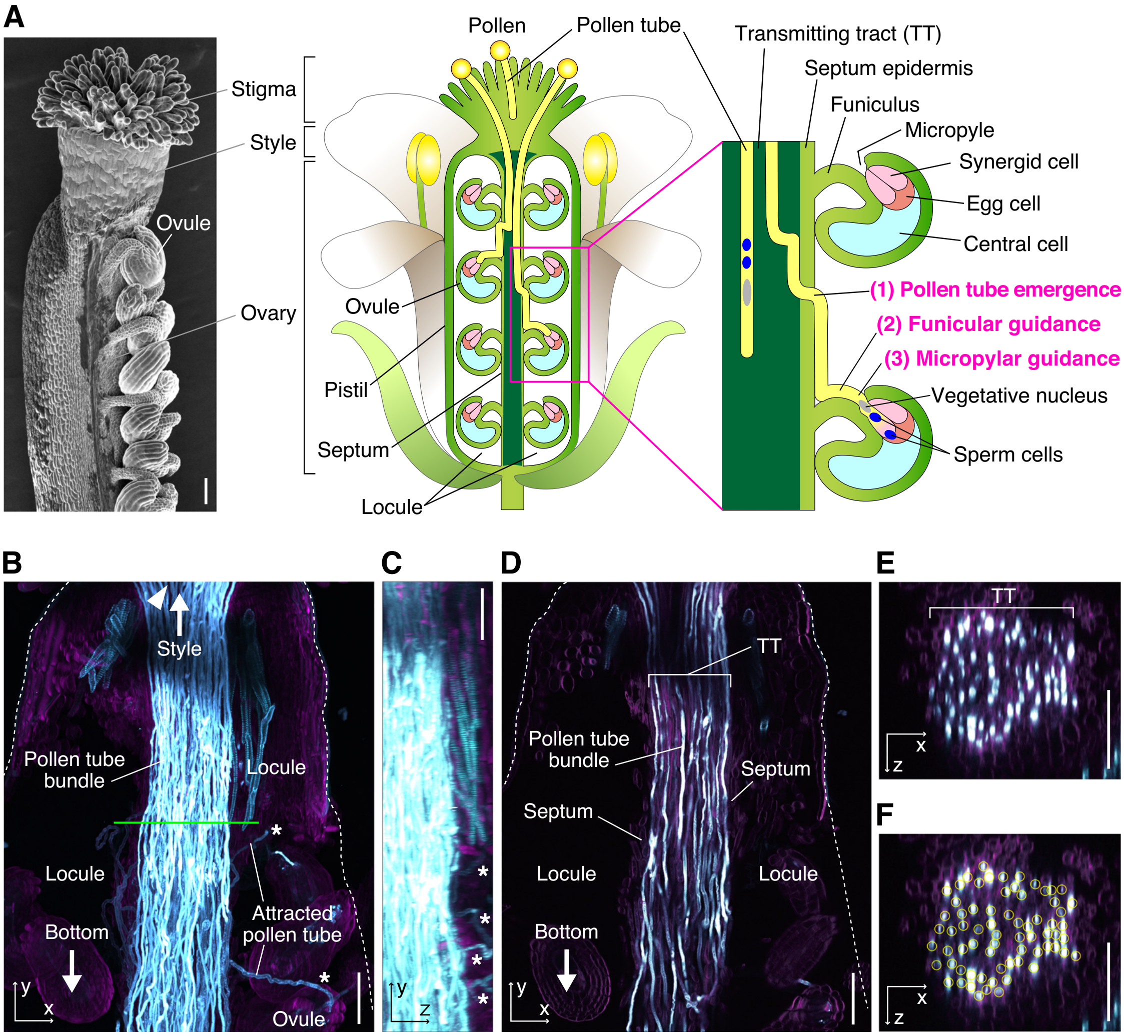

Figure EV1. Reproductive organ structures and processes of *Arabidopsis thaliana*.

(A) (left) Scanning electron microscope image of *A. thaliana* pistil. The right side of the ovary wall has been removed to expose the inner ovules. (right) Schematic representation of the structures of the *A. thaliana* flower and the process of pollen tube guidance inside the ovary. A flower has one pistil in the center, which is formed by fusing the carpels. The fused carpels form two locules, which harbor around 20–30 ovules each in a vertical arrangement inside the locule. Merged region forms a septum harboring the placentae, from where a stalk-like structure funiculus connects each ovule. When pollen lands on the stigma, pollen germinates in the pollen tube. Pollen tube penetrates inside the stigma and enters the style connected to the transmitting tract (TT) in the ovary. Pollen tube guidance after entering the TT was divided into three steps in this study: (1) pollen tube emergence from the TT into a locule (pollen tube emergence), (2) pollen tube guidance from the surface of the septum to the funiculus (funicular guidance), and (3) pollen tube guidance from the funiculus to the micropyle (micropylar guidance). After micropylar guidance, the pollen tube enters the micropyle of the ovule and releases sperm cells in the synergid cells, and then double fertilization with the egg cell and central cell occurs. **(B–F)** Pollen tubes in the TT. Wild-type pistil pollinated with wild-type pollen collected at 18 h after pollination was fixed and cleared by 1 N sodium hydroxide. Pollen tubes in the pistil were stained with aniline blue. **(B)** The *xy*-maximum projection image of the ovary in the maximum pollination. Fluorescent signals of pollen tubes and ovary are shown as cyan and magenta. **(C)** The *yz*-maximum projection image of **(B)**. **(D)** Vertical optical section of **(B)**. Pollen tubes inside the TT of the optical *xy* section are shown. **(E)** Optical cross-section of **(B)** generated by 1-μm steps with 123 planes. The location of the cross-section is indicated by a green line in **(B)**. **(F)** Pollen tubes in the TT of **(E)** were counted by the multi-point tool in the Fiji software. The 69 pollen tubes in the TT are shown as yellow circles. Data Information: **(B–D)** Arrows indicate the directions of style and bottom of the pistil. Arrowhead indicates the end of the style. **(B, C)** Asterisks show the attracted pollen tubes to the ovule located in the back of pollen tube bundle. **(B, D)** White dotted lines show the ovary wall. TT, transmitting tract. **(B–F)** Scale bars, 50 μm.

© The Author(s)

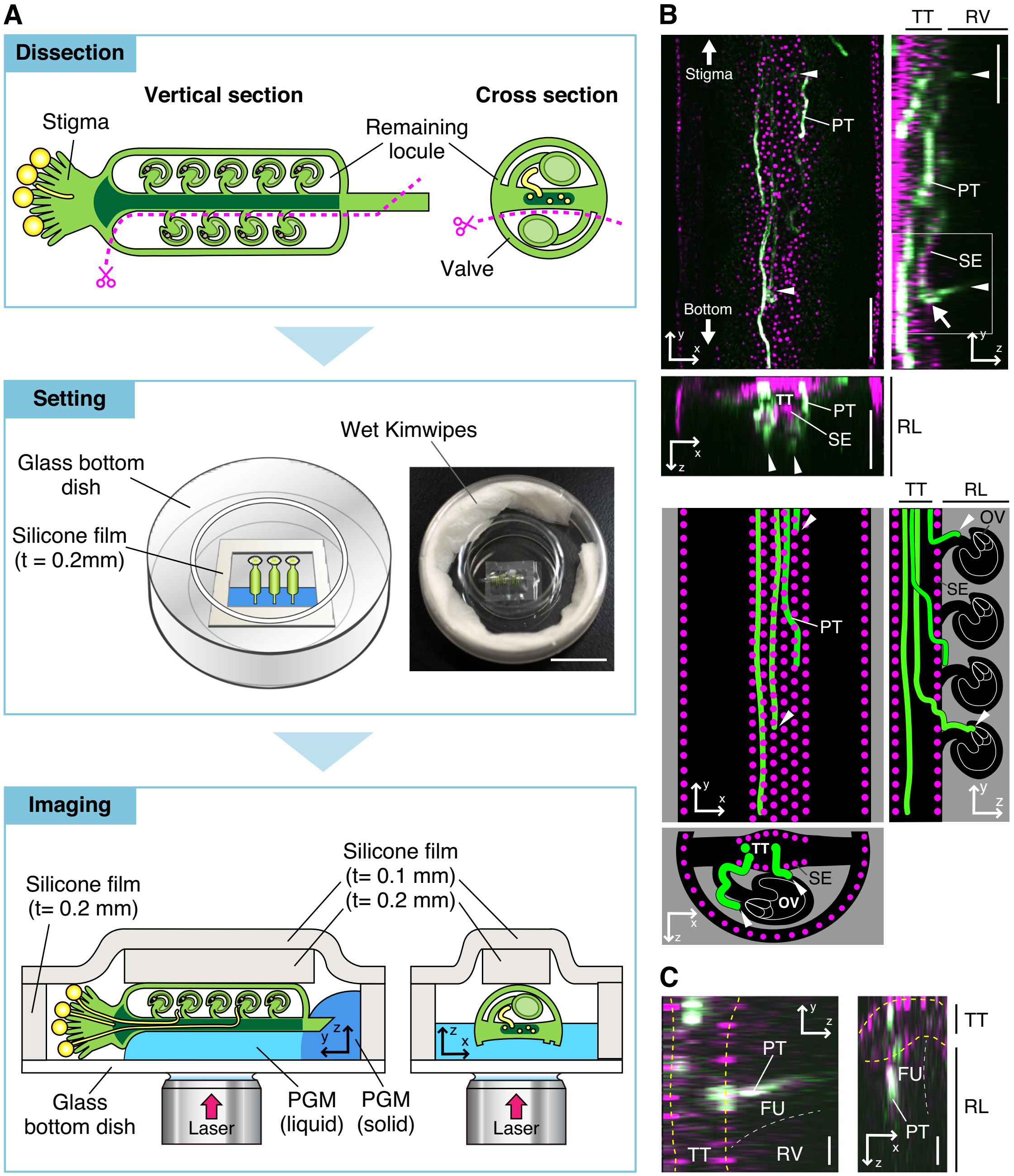

Figure EV2. The single-locule method.

(**A**) Schematic representation of sample preparation of the single-locule method. Pollinated pistils were cut and placed on the mold made with solid pollen germination medium (PGM) in the silicone film at the center of the glass bottom dish. One locule was removed without injuring the septum surface to observe pollen tube guidance inside the transmitting tract (Dissection). To maintain high humidity, wet Kimwipes were placed in the glass bottom dish, and sealed with Parafilm (Setting). These procedures must be carried out quickly and under constant temperature control to prevent tissue damage. A pistil was placed horizontally on the PGM in a silicone frame for observation by an inverted microscope (Imaging). The bottom side with the locule removed was filled with liquid PGM by capillary action. To prevent the pistils from moving and drying, silicone films were placed on both the pistil and silicone flame. Two-photon imaging was performed by the direction of the removed locule. (**B**) Live imaging of the pollinated pistil by two-photon microscopy under the single-locule method. The *xy*-, *xz*-, and *yz*-projection images are shown. Pistil from *HDG11p::NLS-YFP* was pollinated with mTFP1 expressing pollen. Epidermal nuclei and pollen tube in an ovary were labeled with YFP (magenta) and mTFP1 (green), respectively. The top is the stigma side. Schematic representation is also shown at the bottom. Arrowheads indicate attracted pollen tubes toward the ovule. The arrow indicates the point of pollen tube emergence. (**C**) *yz*- and *xz*-optical slice images are shown of (**B**) (white boxes). Fluorescent signals derived from the septum epidermis and funicular autofluorescence are shown as yellow and white dotted lines, respectively. PT pollen tube, OV ovule, SE septum epidermis, RL remaining locule, TT transmitting tract, FU funiculus. Data Information: Scale bars, 1 cm (**A**), 100 μm (**B**), and 20 μm (**C**).

 © The Author(s)

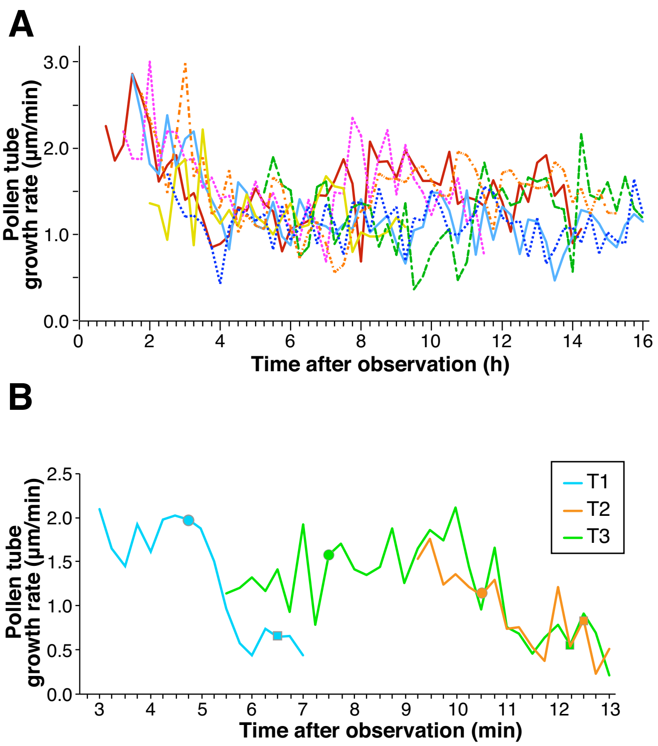

Figure EV3. Pollen tube growth rate in the wild-type (WT) pistils under the single-locule method.

(**A**) Pollen tube growth rate in the transmitting tract (TT) of the maximum pollinated WT pistil. The pollen tube growth rate of 7 non-emerged pollen tubes in a TT shown in Fig. 1B. Maximum intensity *xy*-projections with images taken at 15-min intervals were used for the analysis. See also Movie EV1A. (**B**) The growth rate of three emerged pollen tubes in a TT of the WT pistil with limited pollination shown in Fig. 3B. Maximum intensity *yz*-projections with images taken at 15-min intervals were used for analysis. Filled circles and squares show the time points of the pollen tube attachment to the SE and that of pollen tube emergence, respectively. See also Fig. 3C and Movie EV2C.

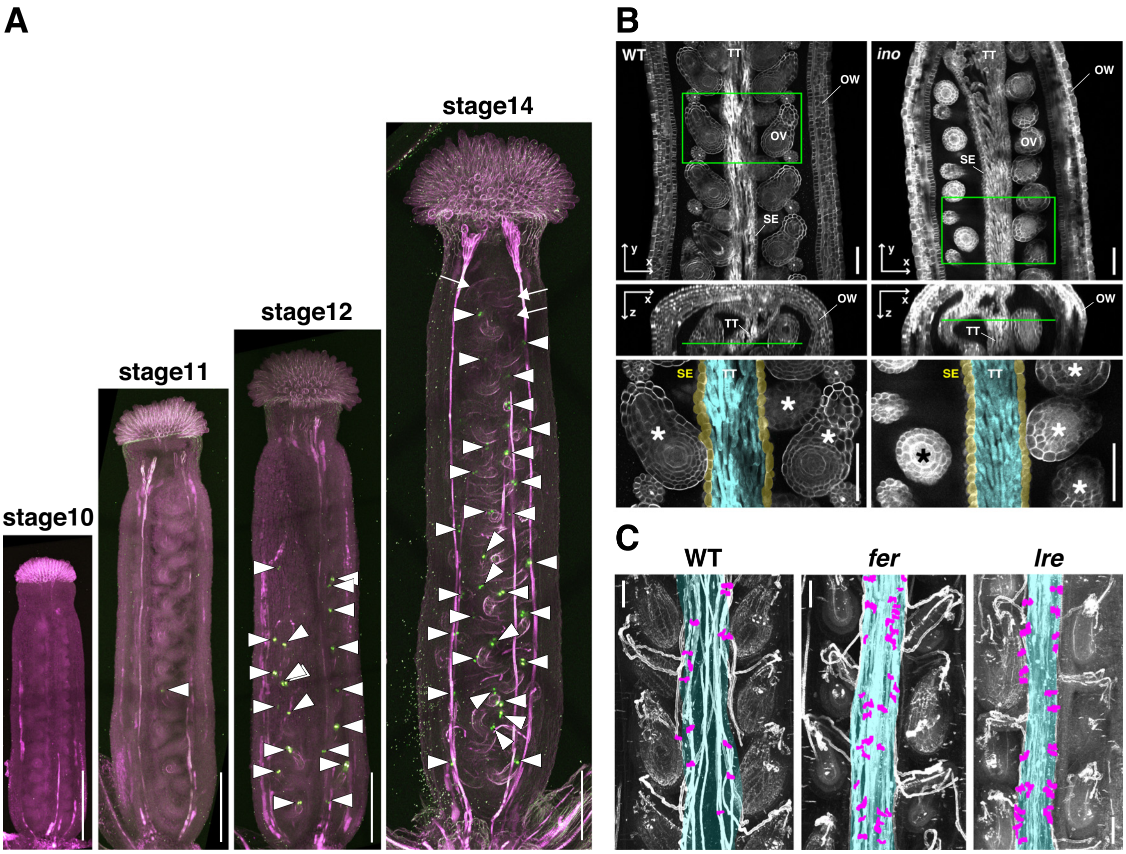

Figure EV4. The internal structure of a transparent pistil.

(**A**) Floral stage-dependent ovule development in the wild-type (WT) pistil. Pistils from stage 10 with petals reaching the length of the lateral stamens to stages 13–14 with opening flower (Smyth et al, 1990) were analyzed. Unpollinated pistils from *FGR8.0* were cleared by ClearSee and observed by two-photon excitation microscopy (2PEM) with 960 nm excitation. Maximum intensity projections for *xy*-projection images were generated from 25–37 z-stack images with 10-μm intervals. Autofluorescence of the pistil is shown in magenta. Arrowheads show the GFP expression in the synergid cells driven by the *MYB98* promoter of the *FGR8.0* construct. Arrows show the ovule without GFP signal in the synergid cells. (**B**) Cell wall-stained clearing WT and *ino* unpollinated pistils. Optical *xy*- and *xz*-sections were generated by 2-μm steps with 101 planes. Magnified images (green box/line) are shown at the bottom. Septum epidermis and transmitting tract (TT) are colored overlay with yellow and cyan. (**C**) Pollen tube emerging points in the WT, *feronia* (*fer*), and *lre* mutant ovaries at 24 h after pollination (HAP). Pollen tubes stained with aniline blue dye and pistil autofluorescence are shown in cyan and gray, respectively. The emerging point on the septum epidermis of each pollen tube is shown as magenta on the *xy*-projection images. SE septum epidermis, TT transmitting tract, OV ovule, OW ovary wall. Scale bars, 200 μm (**A**), and 50 μm (**B**, **C**).

 © The Author(s)

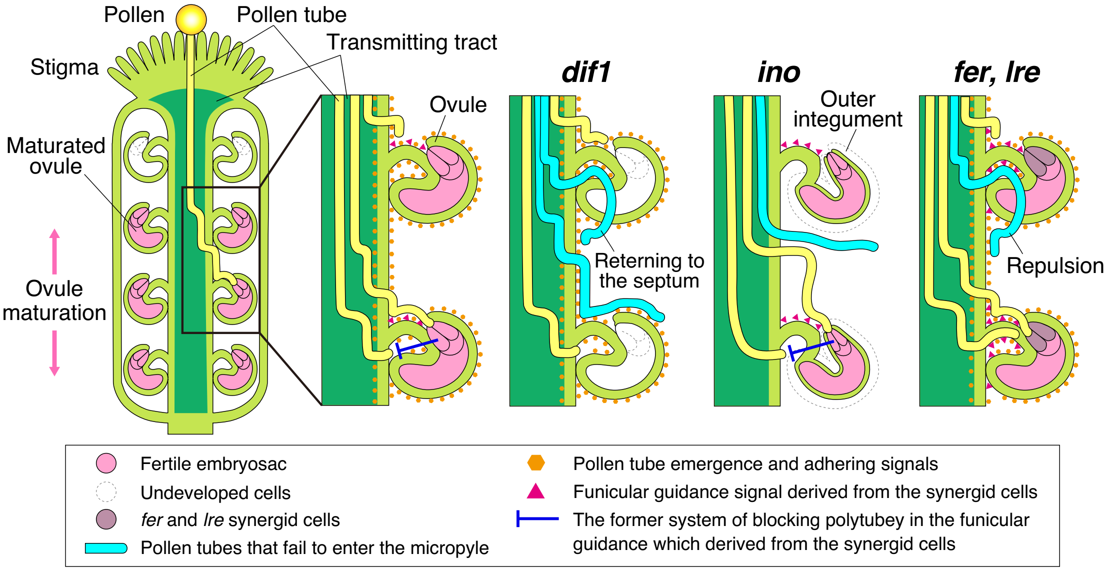

Figure EV5. Model of one-to-one pollen tube guidance in the mutant ovaries.

When the number of pollen tubes is limited, they are preferentially attracted to the ovule located in the lower center of the ovary, depending on the ovule maturation. The pollen tube emergence signal was derived from ovular sporophytic cells. This signal affects the attachment of pollen tubes to the maternal surfaces, which causes a decrease in growth rate and pollen tube emergence into a locule. The emerged pollen tube elongates with attachment to the septum surface and receives funicular guidance signals in a spatially restricted region of the funiculus. The signals derived from the sporophytic and gametophytic cells prevent multiple pollen tube attractions (polytubey blocks) in funicular guidance. In the *dif1* ovary, the pollen tube emergence signal from the ovular outer integument was normal, whereas the gametophytic cell-dependent polytubey block was impaired. In the *ino* ovary, which lacks pollen tube emergence and adhesion signals, a few lucky floating pollen tubes arrive at the micropyle because the signals derived from gametophytic cells function normally. Multiple pollen tubes are attracted along different paths on the funiculus in the *fer* and *lre* ovaries because the gametophytic-dependent attractive funicular guidance signal is increased and/or expanded, or the hypothetical repulsive signal decreases and/or narrows. Pollen tubes that failed to enter the micropyle returned to the septum in the *dif1*, *fer*, and *lre* ovaries. Signals derived from gametophytic cells were impaired in *dif1*, *fer*, and *lre* mutants, which causes polytubey.
